# Modulation of MAA-induced apoptosis in male germ cells: role of Sertoli cell P/Q-type calcium channels

**DOI:** 10.1186/1477-7827-3-13

**Published:** 2005-04-19

**Authors:** Fortunata Barone, Salvatore Aguanno, Angela D'Agostino

**Affiliations:** 1 Biotechnology Unit Casaccia Research Center, ENEA, 00060 Rome, Italy; 2 Department of Histology and Medical Embryology, University of Rome "La Sapienza", 00161 Rome, Italy

## Abstract

Spontaneous germ cell death by apoptosis occurs during normal spermatogenesis in mammals and is thought to play a role in the physiological mechanism limiting the clonal expansion of such cell population in the male gonad. In the prepubertal rat testis, the most conspicuous dying cells are pachytene spermatocytes, which are also the primary target of the apoptosis experimentally induced by the methoxyacetic acid (MAA). Since we have recently reported that Sertoli cells, the somatic component of the seminiferous epithelium, regulate not only germ cell viability and differentiation but also their death, we have further investigated the mechanism involved in such a control.

In this paper we have used the protein clusterin, produced by Sertoli cells and associated with tissue damage or injury, as indicator of germ cell apoptosis in rat seminiferous tubules treated with MAA in the presence or in the absence of omega-agatoxin, a specific inhibitor of P/Q type voltage-operated calcium channels (VOCC's). We performed both a qualitative analysis of clusterin content and germ cell apoptosis by immunofluorescence experiments and a quantitative analysis by in situ end labelling of apoptotic germ cells followed by flow cytometry. The results obtained demonstrate that Sertoli cells modulate germ cell apoptosis induced by methoxyacetic acid also throughout the P/Q-type VOCC's.

## Background

Spermatogenesis in mammals is a complex process dependent upon the hormonal stimulation as well as the dynamic interactions between the Sertoli cells, the somatic component of the seminiferous epithelium, and the germ cells. In fact, in the mammalian testis germ cells, differentiating from spermatogonia to mature spermatozoa, are in close contact with Sertoli cells which supply the nutrients and the hormonal signals essential for successful spermatogenesis. Spontaneous death of germ cells occurs normally during spermatogenesis by the process of apoptosis [1], leading to a loss of up to 75% of the potential number of spermatozoa [2], probably as a physiological mechanism limiting the clonal expansion of germ cells and the spermatozoa release. Such a process has stimulated a number of studies in vivo and in vitro by investigators working in the area of male reproductive endocrinology and toxicology [3-5], with the aim to define the cellular and molecular mechanisms of both spontaneous and toxicant-induced germ cell apoptosis. Among the most commonly toxicants inducing injuries of the male reproductive system [6-8] the 2-methoxyethanol glycol (2-ME), a major bioproduct of the paint industry, causes severe testicular lesions in many mammalian species, including men [9]. By using the methoxyacetic acid (MAA), the proximate toxic metabolite of the 2-ME, germ cell death can be induced in vitro in seminiferous tubule cultures [10]. In 18–21 days old rats, that have not yet completed the spermatogenetic process, the MAA-induced cell death concerns a large proportion of pachytene spermatocytes. We have recently shown that such MAA-induced apoptosis is significantly prevented by co-treatment with nifedipine and ω-conotoxin [11], which block, respectively, L-type and N-type voltage-operated calcium channels (VOCC's). Ca^++ ^channels in many different cell types activate upon membrane depolarisation and mediate Ca^++ ^influx in response to action potentials and sub-threshold depolarising signals. Ca^++ ^entering the cells through VOCC's serves as second messenger of electric signalling, initiating intracellular events such as contraction, secretion, synaptic transmission, and gene expression.

L-type and N-type VOCC's are present on the Sertoli cell plasma membrane [12] and mainly localised at the level of contact surface between Sertoli cells and pachytene spermatocytes in the adluminal compartment of the seminiferous epithelium [12]. Such calcium channels are responsible for the substantial Ca^++ ^influx in rat Sertoli cells [13] and play a role in laminin-dependent [Ca^++^]_i _raise in Sertoli cells and in Sertoli cell secretory process [14,15].

Besides L-and N-type VOCC's, on Sertoli cell plasma membrane is present a third type of Ca^2+ ^channels, the P/Q type [12], specifically blocked by the ω-agatoxin IVA, a peptide isolated from the venom of Agenolanopsis aperta. These channels are localised in the plasma membranes of Sertoli cells adjacent to the basal lamina, at the level of the blood-testis barrier, which selects the substances that can reach the lumen of the seminiferous tubule. P/Q type channels have been shown in neurons where they are primarily responsible for Ca^2+ ^entry and subsequent release of fast neurotransmitters at synapses [18]. Furthermore, several endocrine cell types express these particular Ca^2+ ^channels, still often believed to be expressed only in the nervous system. Pancreatic cells [19], pituitary cells [20], adrenal medullary chromaffin cells [21], as well assmall cell lung carcinoma cells [22] and insulin secreting cell lines [23] express P/Q type Ca^2+ ^channels that participate in the control of their secretory activity.

Clusterin is a ubiquitously expressed heterodimeric glycoprotein that is the major protein produced by cultured rat Sertoli cells [16]. A common theme found in several tissues is the association of clusterin with tissue damage or injury. It has been shown [11,17] that, in MAA-induced apoptosis, Sertoli cell-derived clusterin is very early accumulated in the cytoplasm of dying germ cells at a specific stage of differentiation, i.e. pachytene spermatocytes.

In the present study we used the protein clusterin as a marker of apoptosis to verify whether Sertoli cell P/Q-type VOCC's are involved in the modulation of MAA-induced germ cell death. The presence of VOCC's in the seminiferous epithelium only on Sertoli cell plasma membrane and not on germ cells is very intriguing. Which is the function of such channels in spermatogenesis?

Because of their role in mediating exocytosis and of their peculiar localisation at the level of blood-testis barrier, we would investigate whether P/Q type channels, as well as L- and N-types, were involved in the apoptotic process of germ cells, modulating Sertoli cell response to MAA injury, clusterin secretion and accumulation in pachytene spermatocytes and consequently germ cell death.

## Methods

### Chemicals

ω-agatoxin was purchased from Peptide Institute, Louisville, KY. ω-conotoxin GVIA was purchased by Bachem, UK. Other chemicals, unless otherwise specified, were of the purest grade available from Sigma (St Louis, MO).

### Animals

Male Wistar rats were used in all experiments. Animals were housed in accordance with the guidelines for animal care of University of Rome "La Sapienza" and were killed humanely by asphyxiation with CO_2 _before organ removal. The testes were excised from immature Wistar rats aged from 18 to 21 days, where the spermatogenesis is still uncompleted, washed and immediately processed after excision as described below.

### Sertoli cell in vitro cultures

Sertoli cell monolayers were prepared from the testes of 18–21-day-old Wistar rats according to previously described methods [11,12]. Briefly, after excision, testes were mechanically decapsulated, reduced in small fragments and resuspended in equal volume of Eagle's Minimum Essential Medium (MEM) (Life Technologies, Inc, Grand Islands, NY). Fragments were digested with 0.25 % trypsin (Difco Laboratories, Detroit, MI) 10 μg/ml DNAase I (Roche Molecular Biochemicals, Mannheim, Germany) for 30 min at 32°C, then washed with Hank's Balanced Salt Solutions (HBSS) (Sigma, St Louis MO) and digested with HBSS supplemented with 0.1 % collagenase A plus 10 μg/ml DNAase I (Roche Molecular Biochemicals, Mannheim, Germany) to remove interstitial tissue and peritubular cells. The cell suspension was then centrifuged at 15 × g for 5 min. Pellets were resuspended 1:10 in serum-free MEM with Earle's salt's (Life Technologies, Inc, Grand Islands, NY). Cells were then plated in 3.5 cm Petri dishes and incubated at 32°C in a controlled atmosphere of 95 % air and 5 % CO_2_. On day 3 of culture, cell monolayers were subjected to hypotonic treatment [14] to completely remove endogenous germ cells and were allowed to recover for 24 hours. Experiments were performed on day 4 of culture.

### Treatment with MAA

A 500 mM stock solution of MAA >97 % pure (Fluka, CH9471 Buchs SG, Schweiz) was prepared by dilution in culture medium and the pH was adjusted to 7.4 with 1 N NaOH. This MAA stock was then diluted to a final concentration of 5 mM in the culture medium.

### Treatment with calcium channel blockers

Stock solutions were prepared with appropriate vehicles (H_2_O, 100 % ethanol or 100 % DMSO) according to their solubility. The final concentrations of ethanol and DMSO in the cultures were ≤0.1 %. Vehicle-only controls were included in all experiments. No differences between the medium-only controls and the vehicle-only controls was observed during the experiments.

### Seminiferous tubule in vitro cultures

Testes from 18–21 day-old rats were decapsulated and digested under gentle shaking at room temperature in MEM containing 1 mg/ml collagenase A (Roche Molecular Biochemicals, Mannheim, Germany). After dispersion of the interstitium the tubular mass was rinsed in MEM, then stretches of tubules were dissected by means of sharp needles and carefully transferred to 3.5 cm culture dishes in 300 μl of medium. The tubules were incubated for 10 min at 32°C in a humidified chamber under an atmosphere containing 5 % CO_2_. At the end of the incubation time, the medium was replaced by 600 μl of medium to be exposed to different treatments.

### SDS-PAGE and immunoblotting

Cultured Sertoli cells were scraped off the culture dishes, pelleted and washed with PBS. Pellets were resuspended in lysis buffer (150 mM NaCl, 1 % SDS, 20 mM Tris-HCl, 1 mM EDTA, mammalian Protease Inhibitor Cocktail (Sigma, St Louis, MO), pH 8), and incubated 10 min at room temperature. Samples were then centrifuged at 10, 000Xg for 10 min. Samples containing 20–40 μg protein were loaded onto 10 % Tris-Glycine Bis-Acrylamide gels, run under reducing conditions and blotted onto 0.45 μm nitrocellulose membrane (Novex). After an overnight block with 5 % (v/v) skimmed milk in PBS, the blots were immunostained for 1.5 hr with goat polyclonal antibody raised against a peptide mapping at the carboxy terminus of clusterin of mouse origin (500 ng/ml) (Santa Cruz Biotechnology Inc.). Following incubation in alkaline phosphatase-conjugated rabbit anti-goat IgG (Sigma, St Louis, MO) (1:1000), immunoreactive bands were detected by enhanced chemiluminescence methodology using ECL-Plus, according to manufacturer's instructions (Amersham Biosciences Europe GmbH). All antibodies were diluted in 2 % skimmed milk in PBS (v/v) and the blot was washed extensively with PBS between each stage. Immunoblots were scanned and digitised images were quantitatively analysed by densitometry with AIDA 2.0 program.

### Immunoprecipitation experiments

Immunoprecipitation experiments were carried out to examine whether the VOCC's block of MAA- treated cultured Sertoli cells could modulate clusterin secretion. Sertoli cells were labelled in culture with 50 μCi/ml of ^35^S-methionine (NEN Life Science Products, spec. activ. 1175 Ci/mmol) for 6 hrs in the presence of 5 mM MAA plus the ω-agatoxin (1 μM). At the end of the incubation, the media were removed and processed as described. For immunoprecipitation, we used protein-GSepharose beads (Amersham Pharmacia Biotech) which were first resuspended in RIPA buffer (150 mM NaCl, 0.1 % SDS, 0.5% sodium deoxycholate, and 1 % NP40), washed twice and then incubated for 3 hr with a goat polyclonal antibody raised against a peptide mapping at the carboxy terminus of clusterin of mouse origin (500 ng/ml) (Santa Cruz Biotechnology Inc.), which was used for immunocytochemistry, as well. Following several washes with RIPA buffer, beads were incubated with Sertoli cell culture media overnight at 4°C with continuous agitation. After 1000Xg centrifugation for 1 min and supernatant removal, pellets were washed several times and then resuspended in Laemmli sample buffer (20 % glycerol; 10 % β-mercaptoethanol; 5 % SDS; 0.2 M Tris-HCl, pH 6.8; and 0.4 % bromophenol blue). The samples were boiled for 5 min and run on a 12% SDS-polyacrylamide gel followed by fluorography. Digitised images were quantitatively analysed by densitometry with AIDA 2.0 program.

### Immunolocalisation of clusterin in seminiferous tubule cryosections

The immunohistochemical localization of clusterin protein was performed in cryosections (6 μm) of seminiferous tubules cultured in the presence of MAA, MAA plus ω-agatoxyn or withouth any substance added (control). Slides were first fixed with 4 % paraformaldehyde for 10 min at 4°C, washed twice with PBS and pre-incubated with PBS plus 4 % BSA for 20 min. at room temperature. Sections were then treated with 0.1 % Triton and 0.1 % sodium citrate on ice for 5 min and stained with a goat polyclonal antibody raised against a peptide mapping at the carboxy terminus of clusterin of mouse origin (500 ng/ml) (Santa Cruz Biotechnology Inc.), for 60 min at 4°C in a close, humidified chamber. Then, the cryosections were washed twice with PBS plus 1% BSA and incubated with a FITC-conjugated rabbit anti-goat secondary antibody (Sigma, St Louis, MO), washed with PBS and photographed by a Zeiss fluorescence microscope with an Axiocam imaging system.

### Apoptosis detection of germ cells in seminiferous tubule cryosections

Apoptosis was detected in seminiferous tubule cryosections (6 μm) by using an apoptosis detection system, the In Situ Cell Death Detection Kit (Roche Molecular Biochemicals, Mannheim, Germany). The system end-labels the fragmented DNA of apoptotic germ cells using modified terminal deoxynucleotidyl transferase-mediated dUTP nick end-labeling (TUNEL) assay. It consists in the incorporation of biotynylated nucleotides at the 3'-OH DNA ends, using the enzyme terminal deoxynucleotidyl transferase (TdT). The assay was performed according to manufacturer's instructions. Briefly, sections were rehydrated, washed in PBS and incubated at 37°C for 1 hr with a TUNEL reaction mixture. This allows direct detection of fragmented DNA in apoptotic cells with fluorescein-12-dUTP by means of the enzyme TdT. Negative controls were processed in an identical manner, except that TdT was omitted. As positive controls, sections from control seminiferous tubules were treated with DNAase I for 10 min before performing the end labeling reaction. After the necessary washes, sections were covered with glycerol and glass coverslips.

### Flow Cytometry

For quantitative analysis of germ cells apoptosis levels, cellular suspension prepared from seminiferous tubules was analyzed by flow cytometry using an apoptosis detection system (Roche Molecular Biochemicals, Mannheim, Germany). Seminiferous tubules cultured cultured in the presence of MAA, MAA plus ω-agatoxyn or withouth any substance added (control) were digested with 0.25 % trypsin and 0.02 % EDTA and testicular cell suspension was permeabilized with a permeabilization solution (0.1 % Triton 100X in 0.1 % sodium citrate) for 2 min on ice. Then cells were resuspended in 50 μl of TUNEL reaction mixture or label solution as negative control for 60 min at 37°C, according to manufacturer's instructions. The cells were resuspended in a final volume of 500 μl in PBS and analyzed with a Coulter Epics XL flow cytometer (Beckman Coulter).

For detection of clusterin levels, cell suspension from seminiferous epithelium was washed once with cold PBS plus 1 % BSA before incubation with antibodies. Then, the cells were fixed with 1 % cold paraformaldehyde for 5 min, whereas 0.1 % Triton and 0.1 % sodium citrate in PBS was used for permeabilization. For detection of intracellular clusterin, samples were then incubated overnight at 4°C with a goat polyclonal antibody raised against a peptide mapping at the carboxy terminus of clusterin of mouse origin (500 ng/ml) (Santa Cruz Biotechnology Inc.). Thereafter, cells were washed twice with PBS plus 1 % BSA and incubated 30 min in ice with a FITC-conjugated rabbit anti-goat secondary antibody, washed twice with PBS plus 1% BSA, and analysed. Cells were gated by using forward versus side scatter to exclude dead cells and debris. Fluorescence of 10^4 ^cells/sample was acquired in logarithmic mode for visual inspection of the distributions, and in linear mode to quantify the expression of the relevant molecules by calculating the mean fluorescence intensity. Besides the morphological analysis, DNA was stained with propidium iodide for ploidy analysis to evaluate the purity of each germ cell fraction, as previously described [24].

### Statistics

Student's t test was used for statistical comparison between means where applicable. The statistic software used was the SPSS version 9.0 (Chicago, Illinois).

## Results

### Effects of MAA and ω-agatoxin in rat Sertoli cells in culture

**Figure 1:**
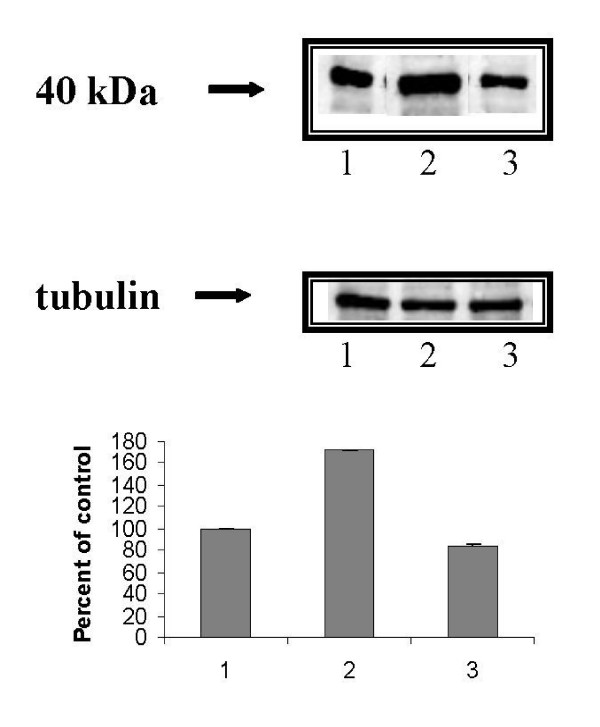
**(Top panel) **Western blotting of rat Sertoli cell lysates treated with polyclonal antibodies raised against clusterin. Representative data of n = 3 independent experiments. **(Lower panel) **Quantification of immunoblot by densitometry. The amount of 40-kDa was quantified by integrating the volume as described under "Methods". Results are expressed as mean +/- SEM of three determinations. 1. control; 2. MAA; 3. MAA plus ω-agatoxin (p < 0.001).

**Figure 2:**
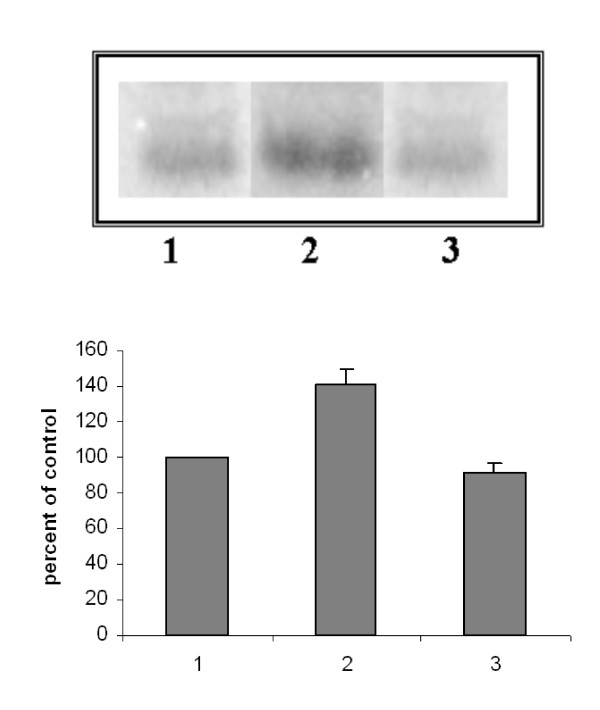
**(Top panel) **SDS-PAGE of immunoprecipitated culture medium from rat Sertoli cells labelled with 35S-methionine. Representative data of n = 3 independent experiments. **(Lower panel) **Quantification of fluorography by densitometry. Results are expressed as mean +/- SEM of three determinations. 1. control; 2. MAA; 3. MAA plus ω-agatoxin (p < 0.001).

In order to use the clusterin as marker of P/Q-type VOCC's-modulated apoptosis it was necessary to verify whether intracellular clusterin level, which is stimulated by MAA in cultured rat Sertoli cells [17,11], was modulated by P/Q-type Ca^2+ ^channels. With the aim to answer this question, Sertoli cells from 18–21 day-old Wistar rats were cultured for 6 hrs with any drug added (control) or in the presence of 5 mM MAA and of 5 mM MAA plus the specific inhibitor of the P/Q-type Ca^2+ ^channels, the ω-agatoxin (1 μM). The samples were analysed by SDS-PAGE followed by Western blot and immunostaining with polyclonal antibodies raised against clusterin. Fig. 1 shows that the MAA-increased amount of clusterin was considerably reduced in the samples treated with MAA plus ω-agatoxin. Fig. 2 shows a modulation also of the secreted clusterin in the culture medium: Sertoli cells, cultured in the presence of MAA, secrete a major amount of clusterin respect to the control; the block of the P/Q channels by ω-agatoxin reduces such a secretion to the basal values.

### Effects of MAA and ω-agatoxin on germ cell apoptosis in cultured rat seminiferous tubules

With the aim to study the effect of MAA and ω-agatoxin on the apoptosis in the seminiferous epithelium, seminiferous tubules were cultured for 6 and 12 hrs in the presence of MAA (5 mM), and MAA plus ω-agatoxin (1 μM). The controls were performed in absence of any drug added to the culture medium. The time of 6 hrs was chosen because is the minimum time required to identify the clusterin in the cytoplasm of pachytene spermatocytes [11,17], while 12 hrs is the minimum time necessary, in our experimental conditions, to detect apoptotic cells in cultured seminiferous tubules.

At the end of the culture an aliquot of seminiferous tubules were immunostained with antibodies raised against clusterin or processed by TUNEL, in order to visualise, respectively, the effect of MAA and ω-agatoxin on clusterin localisation and on germ cell apoptosis in histologic cryosections. The remaining aliquot of seminiferous tubules was trypsinized in order to quantitatively measure the clusterin content and the apoptosis by the flow cytometry analysis. The clusterin content was evaluated after immunostaining of cells with antibodies raised against clusterin while the apoptosis was determined by TUNEL.

**Figure 3:**
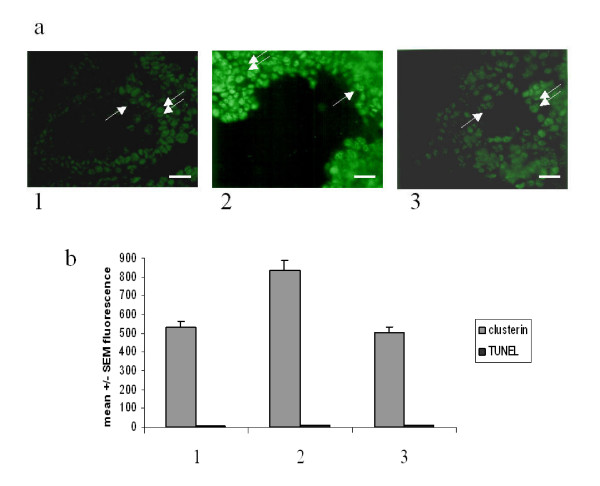
**(Top panel, a) **Immunofluorescence analysis of histologic cryosections of seminiferous tubules cultured for 6 h and immunostained with antibodies raised against clusterin. 1. control (Bar 25x); 2. MAA (Bar 10x); 3. MAA plus ω-agatoxin (Bar 25x). Single arrow indicates pachytene spermatocytes; double arrow indicates Sertoli cells. Representative data of n = 3 independent experiments. **(Lower panel, b) **Quantitative analysis by flow cytometry of testicular cell population prepared from seminiferous tubules cultured for 6 h. Grey bar represents clusterin content (p < 0.001). Black bar represents apoptosis levels determined by TUNEL. Data are expressed as mean +/- SEM of three independent experiments. 1. control; 2. MAA; 3. MAA plus ω-agatoxin.

**Figure 4:**
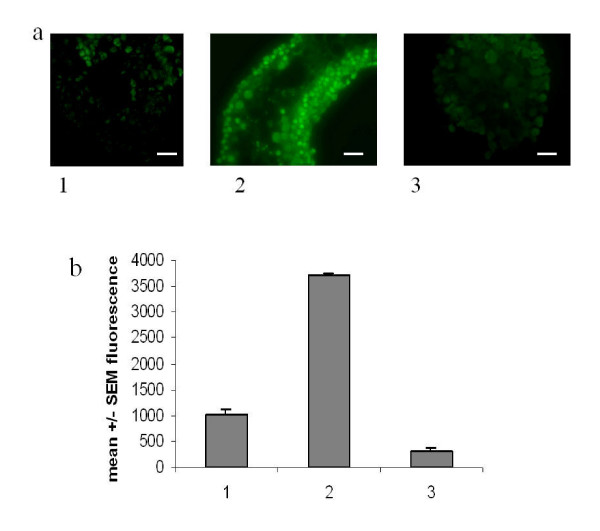
**(Top panel, a) **Immunofluorescence analysis of histologic cryosections of seminiferous tubules cultured for 12 h and processed by TUNEL. 1. control (Bar 20x); 2. MAA (Bar 40x); 3. MAA plus ω-agatoxin (Bar 20x). **(Lower panel, b) **Quantitative analysis by flow cytometry of testicular cell population prepared from seminiferous tubules cultured for 12 h. Bar represents apoptosis levels determined by TUNEL (p < 0.001). Data are expressed as mean +/- SEM of three independent experiments. 1. control; 2. MAA; 3. MAA plus ω-agatoxin.

**Figure 5:**
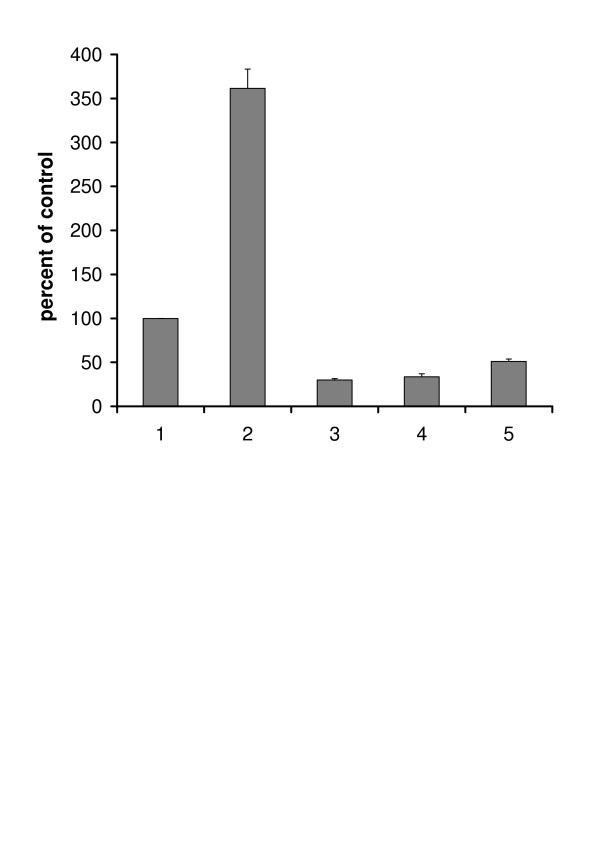
Comparison of VOCC's inhibitors effects on MAA-induced apoptosis of testicular cell population prepared from seminiferous tubules cultured for 12 h. Histograms represent apoptosis levels determined by TUNEL (p < 0.001). Data are expressed as mean +/- SEM of three independent experiments. 1. control; 2. MAA; 3 MAA plus ω-conotoxin; 4. MAA plus nifedipine; 5. MAA plus ω-agatoxin.

The immunofluorescence analysis of the histologic cryosections(Fig. 3) shows that, following 6 hrs of culture, the treatment with MAA not only increases the content of clusterin in Sertoli cells (double arrows) but evidences the presence of clusterin also in germ cells (single arrows) which is reduced in the presence of ω-agatoxin. In parallel, the quantitative flow cytometry analysis confirmed these results. Following 6 hrs of culture, clusterin content is increased in MAA-treated seminiferous tubules (grey histogram 2) and it is reduced in seminiferous tubules treated with MAA plus the ω-agatoxin (grey histogram 3). Apoptosis is not appreciable (black histograms), in agreement with literature data *in vitro *and *in vivo *[10,11], due to the short time of MAA-treatment. In Fig. 4 the immunofluorescence analysis of seminiferous tubules cultured for 12 hrs shows that the apoptosis of pachytene spermatocytes (single arrows) is enhanced in the presence of MAA and is decreased by the VOCC's inhibitor. Fig. 4 also shows the analysis performed by flow cytometry: ω-agatoxin (histograms 3) does decrease the MAA-induced germ cell apoptosis (histogram 2). Fig. 5 shows a comparison among ω-agatoxin, nifedipine and ω-conotoxin effects on MAA-induced apoptosis. The inhibitory effect due to the block of P/Q type channels appears less pronounced (mean +/- SEM: 51 +/- 2.8) than that of L (nifedipine-sensitive) (mean +/- SEM: 30 +/- 1.4)and N (ω-conotoxin-sensitive) (mean +/- SEM: 33.5 +/- 3.5) type VOOC'S. Expressed as percentage ofinhibition of the MAA response, the block due to ω-agatoxin is of 85%, while nifedipine and ω-conotoxin blocks are of 91% and 90 %, respectively.

## Discussion

A growing body of evidence demonstrates that both spontaneous (during normal spermatogenesis) and increased germ cell death triggered by various regulatory stimuli [25-29] in rats occur via apoptosis. However, the mechanisms by which these proapoptotic stimuli activate germ cell death are not well understood. We have recently proposed a calcium-mediated mechanism underlying spermatocyte apoptosis induced by MAA [11]. It has been shown that in rat seminiferous epithelium Sertoli cells can modulate germ cell apoptosis throughout L-type and N-type VOCC's and that the contacts between Sertoli cells and germ cells are necessary for such a modulation [11].

In the present study we demonstrate that Sertoli cells are involved in MAA-induced germ cell apoptosis also throughout VOCC's blocked by ω-agatoxin, i.e. of P/Q type. The block of such VOCC's inhibits the accumulation of Sertoli cell-produced clusterin in the cytoplasm of germ cells, consequent to the MAA treatment, and prevents pachytene spermatocyte apoptosis. The data presented here, however, show that, from a quantitative point of view, the inhibition of germ cell death is less pronounced respect to that induced by nifedipine and ω-conotoxin, suggesting a synergistic role of P/Q type calcium channels. These results fit well with the peculiar localization of P/Q type calcium channels in the seminiferous epithelium.

The previous identification of P/Q channels on the plasma membrane of Sertoli cells in the zone adjacent to the basal lamina and their involvement in the modulation of protein secretion [12], suggested a role at the level of the blood-testis barrier (BTB), which selects the substances that can reach the lumen of the seminiferous tubule. At the level of BTB the Ca^2+ ^plays a relevant role. It is known that the Sertoli cell barrier can be disrupted and resealed by manipulating [Ca^2+^] in the culture medium [31]. In the rat, the entire process of germ cell development, except for the early phase of spermatogenesis, is segregated from the systemic circulation by the BTB. This means that whatever substance would have to pass through the Sertoli cell cytoplasm to reach the late meiotic and post-meiotic germ cells. P/Q-type channels should represent the first "sentinel"gates of Sertoli cells and could constitute a first inhibitory mechanism respect to L-and N-type channels, which are localized deeply in the seminiferous epithelium, near the lumen of the tubule. Furthermore, the ascertainment that the block of P/Q-type VOCC's inhibits germ cell apoptosis less than L- and N-type should suggest a gradual, spatial control of germ cell death.

The decrease of intracytoplasmic Sertoli cell clusterin and the inhibition of its accumulation into pachytene spermatocytes, following P/Q channels block, suggest that calcium channels control germ cell apoptosis throughout an indirect mechanism. The protective effect of calcium channel blockers against MAA-induced spermatocyte apoptosis is probably mediated by the inhibition of clusterin transfer from Sertoli cells to germ cells. This hypothesis agrees with recent data from literature which demonstrate that the intracellular form of clusterin could be cytotoxic [32-34]. Clusterin accumulation in germ cells could be a selection mechanism for cells secondarily injured and committed to death. On the other hand, being Sertoli cell the direct target of MAA, the injured cell should be not more able to support a further differentiation of germ cells and should limit the number of spermatocytes. This latter hypothesis agrees with recent results obtained in MAA-treated rats where the expression of both Sertoli cell androgen receptor protein and androgen binding protein are significantly altered [35]. It is known, in fact, that the diminution in androgen receptor is concomitant to a decrease of intratesticular androgen levels and is accompanied by significant germ cell apoptosis [7,36]. Altered expression of ABP in transgenic mice is associated with relevant apoptosis of pachytene spermatocytes [37].

Furthermore, in this paper we demonstrate a role of P/Q-type VOCC's specific for the control of male germ cell death. In other cell types, i.e. cultured cortical neurons, exposed to amyloid β protein in order to induce apoptosis, the block of L-type voltage-sensitive Ca^++ ^channels attenuates neuronal apoptosis while the block of N- and P/Q type channels has no effect [38].

Our work confirm and extend previous data showing that the VOCC's localized exclusively on Sertoli cell plasma membrane modulate Sertoli cell response to injuries that could damage germ cell differentiation. Such intratesticular regulatory mechanism controlling germ cell apoptosis adds a further piece of the puzzle concerning the knowledge of the apoptotic program in the testis [39].

## Authors' contributions

FB was responsible for experimental activity. She performed cell cultures, immunofluorescence and flow cytometry analysis, partecipated in the analysis of data and in drafting the manuscript. SA collaborated in the immunofluorescence analysis of seminiferous tubule cryosections and provided valuable suggestions during writing the manuscript. AD was responsible for design and coordination of the study. She analyzed the data and drafted the manuscript. All authors read and approved the final manuscript.
